# Dioctylsulfosuccinate Functionalized NiAl-Layered Double Hydroxide for Sensitive Fenuron Electroanalysis Using a Carbon Paste Electrode

**DOI:** 10.1155/2024/9237309

**Published:** 2024-07-19

**Authors:** Aude Peggy Kameni Wendji, Herve Leclerc Tcheumi, Ignas Kenfack Tonle, Emmanuel Ngameni

**Affiliations:** ^1^Department of Mineral Engineering, School of Chemical Engineering and Mineral Industries, The University of Ngaoundere, P.O. Box 454, Ngaoundere, Cameroon; ^2^Laboratory of Analytical Chemistry, Faculty of Science, The University of Yaounde I, P.O. Box 812, Yaounde, Cameroon; ^3^Department of Environmental Sciences, National Advanced School of Engineering of Maroua, The University of Maroua, P.O. Box 46, Maroua, Cameroon; ^4^Department of Chemistry, Faculty of Science, The University of Dschang, P.O. Box 67, Dschang, Cameroon

## Abstract

Environmental pollution resulting from the use of pesticides such as fenuron poses significant health risks due to the carcinogenic and teratogenic properties of these compounds. There is an urgent need to develop rapid and cost-effective detection methods for quantifying fenuron. In this study, an inorganic-organic composite material was obtained by intercalating sodium dioctylsulfosuccinate (DSS) within the interlayer space of a nickel-aluminum-layered double hydroxide (NiAl-LDH). The pristine and modified LDHs (NiAl-LDH) were characterized using Fourier transform infrared, X-ray diffraction, and thermogravimetric analysis, confirming the successful intercalation of DSS in the mineral structure. The modified LDH was used to elaborate a sensor for detecting fenuron herbicide via differential pulse voltammetry (DPV) employing a carbon paste electrode (CPE). The electrochemical procedure for fenuron analysis consisted of immersing the working electrode in an electrolytic solution containing the appropriate amount of fenuron, followed by voltammetry detection without any preconcentration step. Compared to CPE modified by pristine LDH, the peak current obtained on the organo-LDH-modified CPE was twice as high. The increase in the fenuron signal was attributed to the high organophilic feature of this composite material induced by DSS modification. To optimize the sensitivity of the organo-LDH modified electrode, the effects of several experimental parameters such as pH of the medium and proportion of the modifier in the paste on the stripping response were examined. Linear calibration curves were obtained for the fenuron concentrations ranging from 0.5 × 10^−6^ to 1 × 10^−6^ mol.L^−1^ and 1 × 10^−6^ to 5 × 10^−6^ mol.L^−1^. The limit of detection (LOD) calculated on the basis of a signal-to-noise ratio of 3 was found to be 1.8 × 10^−8^ mol.L^−1^ for the low concentration range with a limit of quantification (LOQ) which was 6 × 10^−8^ mol.L^−1^. Furthermore, the interference effect of several inorganic ions and other pesticides potentially affecting fenuron stripping was explored, and the method's applicability was confirmed by determining fenuron levels in a river sample taken from down-town Yaoundé.

## 1. Introduction

Herbicides are frequently utilized in various environments to manage weeds both before and after their emergence. Noncultivated areas such as highways and railroads are also affected by the extensive use of herbicides. Fenuron (1,1-dimethyl-3-phenylurea, referred as FEN) is one of the herbicides that is commonly employed nowadays [1–3]. This phenylurea herbicide is effective in preventing the germination of grasses and broadleaf weeds [4]. However, due to its widespread use and relatively long environmental lifespan, residues of fenuron have been detected in both ground and surface waters [5]. Given the known toxicity of fenuron and its associated concerns regarding mutagenicity and carcinogenicity, these residues cause a very substantial environmental risk to human health and ecosystems [6, 7]. Accurate detection of fenuron in environment, including soil, water, and crops, is crucial for assessing contamination levels in specific areas and determining necessary cleanup measures.

The most common and frequently used method for the detection of pesticide is chromatographic methods [8, 9], but also ultraviolet spectroscopy and spectrophotometry have been employed to analyze organic pollutants [10, 11]. While these techniques have proven successful, they require a lengthy analytical time procedure and numerous pretreatment processes, in addition to exhibiting poor selectivity and sensitivity. Hence, the development of straightforward, inexpensive, and sensitive alternatives for FEN detection remains a serious challenge.

Electroanalytical procedures, particularly when applied to environmental issues such as water pollution, have demonstrated high efficacy in analyzing hazardous contaminants like pesticides. Furthermore, electroanalytical techniques have proven to be a good alternative according to existing methods in the analysis of substances with low detection limits and sensitivity [12, 13]. A variety of materials, including zeolite [14] and tungsten oxide, have been used as modifiers of carbon paste electrode for detection of various pesticides in water amitrole [15, 16] and carbendazim [17] in water. Layered double hydroxides (LDHs) are synthetic layered metal hydroxide materials that have an excess of positive charge in their brucite-like layers. Due to their biocompatibility and swelling properties, these minerals have garnered considerable attention in sensing applications [18, 19]. However, LDHs exhibit poor selectivity and limited adsorption capacities for hydrophobic organic compounds due to their hydrophilic surfaces [19–21]. Despite these appealing characteristics, LDHs have to enhance their selectivity and adsorption capacities and the functionalization of their interlayer space has emerged as an effective strategy.

Many studies related to the functionalization of LDH using the intercalation of organophilic anionic surfactants in the interlayer space have been reported [22–24]. The resulting materials showed a high affinity for hydrophobic contaminants [25, 26] including phenylurea herbicides [27]. However, there remains a gap in research concerning the development of electrodes for amperometric sensor quantification of phenylurea herbicides. In this regard, Siara et al. [14] developed a sensitive electrochemical method for quantifying linuron using a zeolite modified carbon paste electrode. Manisankar et al. [27] developed a polypyrrole modified electrode for isoproturon electrochemical determination. Gonzalez de la Huebra et al. [28] also described the use of a carbon fiber microelectrode to study the electrochemical behaviour and electroanalytical determination of linuron in soil extracts. Wong et al. [29] developed a nickel octylbutoxy-phthalocyanine complex to modify a carbon paste electrode for sensitive and selective electroanalysis of diuron herbicide. Despite the numerous existing phenylurea compounds, few research works reported on the development of an electrode for amperometric sensor quantification of FEN [30, 31]. Therefore, there is a need to develop efficient electrode materials that will enable the fabrication of low-cost electrochemical sensors capable of enhancing the performance of existing FEN electrochemical sensors. To the best of our knowledge, no work has been reported on the exploitation of organo-LDH as an electrode modifier in connection to the electroanalysis of phenylurea compounds such as FEN.

The main goal of this work is to develop an electrochemical sensor for FEN quantification based on a carbon paste electrode modified with an organo-LDH. For this, a nickel-aluminum-based LDH (NiAl-LDH) was synthetized and modified with the organophilic anionic surfactant sodium dioctylsulfosuccinate (DSS) to improve its affinity towards FEN herbicide. Given the sorbent properties of organo-LDH towards FEN (hydrophobic interaction), its application as an electrode modifier was also expected to facilitate the detection of FEN. The proposed sensor, elaborated with the resulting organo-LDH, demonstrates high sensitivity and reliability for real-life practical application.

## 2. Experiment

### 2.1. Chemicals and Reagents

All chemicals and reagents used in this study were of analytical grade and were used without further purification. FEN (98.7%) was supplied by Prolabo. A stock solution of FEN (0.01 M) was prepared in 95% ethanol. Prolabo sodium dioctylsulfosuccinate (DSS) was used as received, and the acetate buffer solution used as the supporting electrolyte was prepared by combining 0.1 M CH_3_COONa and CH_3_COOH (Riedel-de-Han). Al (NO_3_)_3_ and Ni (NO_3_)_2_ were purchased from Sigma Aldrich. For the interference studies, CaCl_2_, MgSO_4_, NaCl, PbCl_2_, and ZnCl_2_ (all from Sigma Aldrich) were used; also, methyl parathion was acquired from Supelco, and a stock solution (0.01 mol.L^−1^) was prepared in ethanol, and glyphosate was purchased from Sigma Aldrich, and a stock solution (0.01 mol.L^−1^) was prepared in deionized water. NaOH and HCl (37%) obtained from BDH and Prolabo, respectively, were used to adjust the pH. All aqueous solutions were prepared using deionized water.

### 2.2. Preparation of LDH and Modified LDH

NiAl-LDH was prepared following the procedure described by Kameni et al. [23, 32]. A mixed salt solution (50 mL), containing nickel nitrate and aluminum nitrate in the molar ratio 3/1, was prepared by dissolving 0.045 mol and 0.015 mol, respectively, in previously boiled deionized water. The mixture was purged with N_2_ to avoid carbon dioxide. Sodium hydroxide (2 M, 25 mL) was added dropwise to the solution while vigorously stirring, maintaining the pH at 10.1 ± 0.5. The resulting solid product was recovered by centrifugation, washed thoroughly with deionized water, and oven-dried for 24 hours at 70°C. The prepared material was labeled NiAl-LDH.

The synthesis of modified LDH, designated as NiAl-DSS, was adapted from a published procedure using the homogeneous coprecipitation method [33]. Briefly, three solutions were prepared in boiled deionized water: one containing a mixture 50 mL of 0.045 mol nickel nitrate and 0.015 mol aluminum nitrate and the other one involving 10 mL of 0.001 M of modifier agent (DSS) and the last one of 25 mL of 2 M sodium hydroxide. These solutions were concomitantly added dropwise into a round bottom flask under stirring and in a nitrogen atmosphere. The pH of the mixture was maintained between 9.5 and 10.5. Resulting suspension was vigorously stirred for 16 hours in a nitrogen atmosphere at 75°C. Later on, it was filtered and the collected solid product was thoroughly washed with deionized water and dried at 70°C for 24 hours.

### 2.3. Characterization

XRD patterns were recorded at room temperature using a classical powder diffractometer (X'PERT PRO/Philips) equipped with a Cu anode (quartz monochromator, *kα*_1_ radiation, *λ* = 1.54056 Å). Thermogravimetric analyses of samples were performed using a TA Q5000 under nitrogen flow rate of 25 mL.min^−1^. About 20 mg of powder sample was placed on the analyzer's thermobalance, which was purged with helium gas. The measurements were taken between 50 and 600°C with a temperature ramp of 10°C.min^−1^, and data were analyzed using the universal analysis 2000 software package. The thermal gravimetric (TG) weight loss curve and the first derivative of this curve (DTG) results were present on each plot. The Fourier transform infrared (FT-IR) spectra were recorded using the KBr method covering the range from 4000 to 500 cm^−1^ with an alpha spectrometer from Bruker Optics. The resolution of the spectra was 4 cm^−1^. A scanning electron microscope (Hitachi TM 4000 plus) equipped with energy dispersive spectroscopy (EDS) was used to investigate the morphology and composition of NiAl-LDH and NiAl-DSS. Images were collected on backscattered electron mode, and the point EDS was collected using an accelerating voltage of 15 kV.

### 2.4. Working Electrode Preparation, Electrochemical Equipment, and Procedures

Carbon paste electrodes (CPEs) were made by combining carbon powder (≤325 µm), binder (mineral oil), and modifier (LDH or organo-LDH particles) in the optimized weight ratio of 68 : 30 : 2. As a control, an unmodified carbon paste electrode (CPE) was made by combining only carbon powder and mineral oil (in the proportion 70 : 30). In Teflon tubes with a 6-mm internal diameter and an electrical contact made of a stainless steel screw and piston, the modified and unmodified paste electrodes were inserted. Prior to the experiment, the electrode surface was polished with a filter paper. In a three-electrode cell setup, the CPE/NiAl and CPE/NiAl-DSS were employed as working electrodes, a Pt wire served as the counter electrode, and a saturated calomel electrode (SCE) as the reference electrode.

The electrochemical measurements were carried out using an Autolab potentiostat connected to a computer and managed by the GPES electrochemical analysis system (Echo Chemie, the Netherlands). Cyclic voltammograms and differential pulse voltammograms were recorded by immersing the working electrode in the electrolytic solution containing appropriate amount of FEN followed by voltammetry detection without any preconcentration step. Precisely, 25 mL of the supporting electrolyte was placed in the voltammetric cell and the required volume of FEN solution was added using a micropipette to obtain the desired concentration of FEN. The solution was then deaerated with nitrogen for 10 min after which, the voltammetric curve was recorded. Prior to the next measurement, the electrode was regenerated by transferring it to a blank acetate buffer solution under stirring, where the trace amounts of FEN totally desorbed after one minute.

## 3. Results and Discussion

### 3.1. Characterization of LDH and Organo-LDH

**Figure 1:**
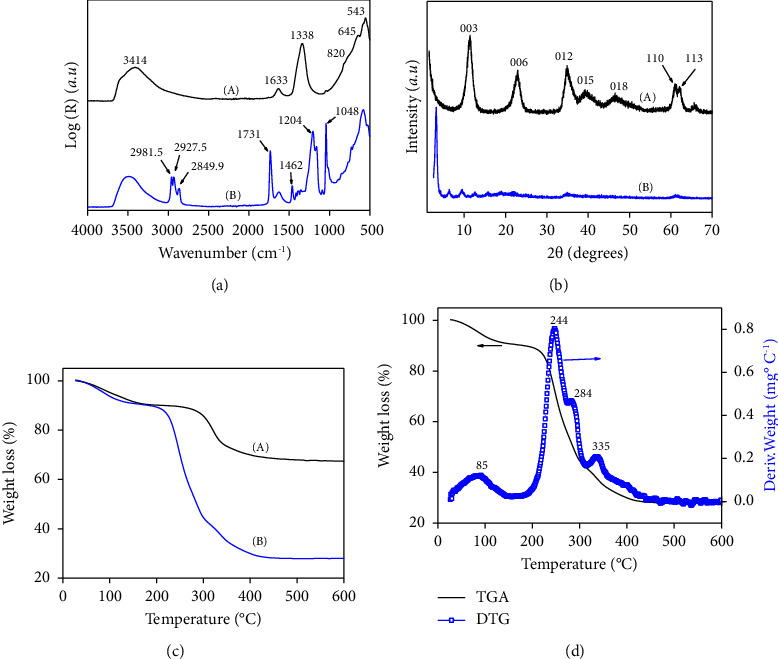
(a) FT-IR spectra of (A) NiAl and (B) NiAl-DSS; (b) Powdered XRD pattern of (A) NiAl and (B) NiAl-DSS; (c) TGA of (A) NiAl and (B) NiAl-DSS; (d) DTG traces of NiAl-DSS.

The FT-IR spectra of NiAl and modified-NiAl are displayed on Figure 1(a). The broad band centered around at 3414 cm^−1^ in the spectrum of NiAl corresponds to the stretching vibration mode of hydroxyl group of LDH and water molecules (intercalated and physically adsorbed) [34, 35]. The bending vibration of these water molecules is reflected in the band at 1633 cm^−1^. The band at 1338 cm^−1^ is attributed to NO_3_^−^ present in the interlayer space to compensate the positive charge of the NiAl layers. The band at 820 cm^−1^ is due to Ni-O bonds, the band at 645 cm^−1^ to NiO_6_ octahedra, and the band at 543 cm^−1^ to AlO_6_ octahedra [36]. Moreover, the absence of bands at 1500, 1360, and 960 cm^−1^ confirm the absence of carbonate ions in the interlayer space [37, 38]. The spectrum of NiAl-DSS showed new bands, including those in the range of 2849 cm^−1^ to 2981 cm^−1^ assigned to aliphatic C-H stretching vibration [37, 39], and bands at 1462 cm^−1^ and 1731 cm^−1^ corresponding to bending vibrations of the aliphatic C-H bonds [36, 40] and presence of carbonyl of ester group [37, 41], respectively. The bands observed at 1204 and 1048 cm^−1^ are due to the antisymmetric and symmetric stretching vibration of S=O bond, respectively [42]. These bands indicate the presence of DSS in the synthesized material. Moreover, the absence of associated bands to nitrates in the spectrum of NiAl-DSS suggests preferential intercalation of DSS ions between the interlayer spaces of NiAl-LDH, consistent with previous reports [43]. The main features of FT-IR spectra of pristine LDH and functionalized LDH are presented as supplementary materials in Table SI 1 for band assignments.

The XRD pattern of the precursor material (NiAl) is shown in Figure 1(b)(A). The characteristic reflection of (003), (006), (012), (015), and (018) planes of a crystalline LDH can be observed. The typical doublet observed at (110)–(113), between 60 and 63°, indicates the good crystallinity of this material and is consistent with other studies [44–47]. The highest peak, located at 2*θ* = 11.12°, corresponds to the (003) plane, associated with an inter-reticular distance (d_003_) with a value of 7.94 Å according to the Bragg relation. This value is slightly lower than that obtained by other authors during the synthesis of a layered double hydroxide with equivalent composition of nitrate ions in the interlayer space (8.23 Å) [48]. Such variations of d_003_ are commonly observed with LDH, due to the level of hydration of LDH [49]. In fact, the hydrophilic properties of interlayer space of LDH facilitate the insertion of water molecules [49]. On the XRD pattern of NiAl-DSS, the characteristic peak of the (003) diffraction plane shifted to the small angles, with a d_003_ value of 27.4 Å. This corresponds to an increase of 19.46 Å indicating the important expansion of the interlayer space after intercalation of DSS (Figure 1(b)(B)). Also, the decrease of peak intensities of those planes in case of NiAl-DSS indicates a very low crystallinity attributed to the delamination of clay layer [50, 51]. The value of interlayer space of organo-LDH depends on the orientation of intercalated anions. In order to evaluate this orientation, the molecule size of DSS ions was estimated using ChemSketch software and was found to be approximately 22 Å (length) and 5 Å (width). If DSS ions are arranged vertically in the interlayer space of NiAl-DSS, and then the theoretical value of the d-spacing after modification is expected to be 29.74 Å (7.94 + 22 = 29.94 Å), while the experimental value obtained (27.4 Å) suggests a pseudo paraffin-type monomolecular arrangement of DSS ions in the interlayer of NiAl-DSS. Thus, from the results of the XRD studies, it could be inferred that the DSS anions were successfully intercalated into the LDH galleries.

The results of thermogravimetric analysis of NiAl and NiAl-DSS are presented in Figure 1(c)((A) and (B)). The TGA curve of NiAl (Figure 1(c)(A)) showed two decomposition steps. The first step was observed between 25° and 170°C with a weight loss of 11.8% which corresponds to the water molecules adsorbed on the external surface of NiAl or in the interlayer surface. The second weight loss observed between 170° and 500°C is due to the dehydroxylation of the metal hydroxide layer [35, 38, 52]. The thermal behaviour of NiAl-DSS (Figure 1(c)(B)) showed multiple thermal events. In the temperature range of 50–150°C, organo-LDHs display lower weight loss than LDH (8.9%) indicative of less free water in their bulk structure probably due to the hydrophobic nature of DSS. The mass losses at 244°C and 284°C (Figure 1(d)) correspond to the decomposition of intercalated DSS [53]. The weight loss at 335°C corresponds to the dehydroxylation of NiAl [23].

**Figure 2:**
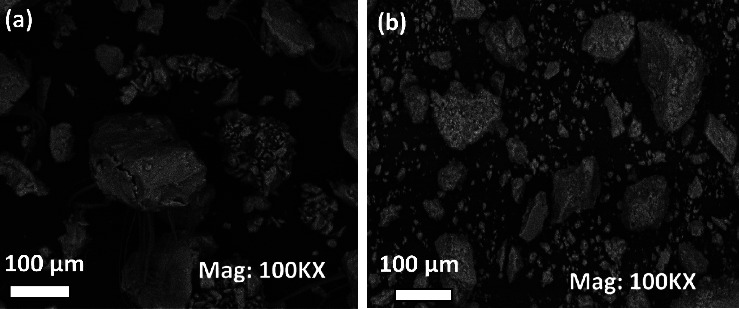
SEM micrographs of (a) LDH and (b) LDH-DSS.

SEM images of LDH and modified LDH (Figure 2) show morphological changes during intercalation of DSS. It can be seen in Figure 2(a) that LDH has hexagonal morphology, covered by small separated particles. The image of LDH treated with DSS indicated the change of morphology. The particles of LDH-DSS are agglomerated (Figure 2(b)). In fact, the organo-LDH particles aggregated easily because the surfactants compensate for the positive charge and thus eliminate the repulsive electrostatic forces between LDH particles [46].

Table 1 lists the energy dispersive spectroscopy (EDS) results of components of LDH and LDH-DSS. As shown in Table 1, the EDS results of LDH shows the presence of the following elements: C, N, O, Na, Ni, and Al. From the EDS spectrum of LDH-DSS, we observed the presence of some amounts of sulfur and the absence of nitrogen. This result suggests that most of nitrate anions in the interlayer space were replaced by DSS through the anionic exchange reaction. This observation is in agreement with the FT-IR result.

### 3.2. Electrochemical Characterization of LDH and Organo-LDH

In order to explore the use of the NiAl-DSS material as a modifier of CPE, preliminary experiments were performed using [Fe(CN)_6_]^3−^ ions as electroactive probe. The electrochemical behaviour of [Fe(CN)_6_]^3−^ ions at bare CPE and modified CPE was recorded upon repetitive scanning by cyclic voltammograms (Figure SI 1). At bare CPE (Figure SI 1a), well reversible cyclic voltammograms with a constant steady state were recorded upon repetitive scanning (5scans). When CPE was modified by NiAl-LDH, the result obtained is illustrated in Figure SI 1b (supporting information). A low accumulation of [Fe(CN)_6_]^3−^ ions during the first scan, due to the effect of nonconductive property of LDH material [32, 35], is observed. Upon multiscan, a gradual increase in the intensities of the signals is observed; this behaviour is due to anionic exchange properties of NiAl-LDH [33, 35]. The peak current reaches its maximum value after 50 cycles, with the current being twice as high as that recorded on bare CPE. A similar behaviour was observed upon modification of CPE with NiAl-DSS (Figure SI 1c) although with a lower accumulation of [Fe(CN)_6_]^3−^ ions. The lower accumulation can be explained by the decrease in the anion exchange capacity of the organo-LDH (NiAl-DSS).

In order to confirm the electron transfer properties of CPE/NiAl-DSS, EIS measurements were performed using CPE, CPE/NiAl, and CPE/NiAl-DSS electrodes in 0.1 M NaCl containing 10^−4^ M [Fe(CN)_6_]^3−/4^ as redox probe (Figure SI 1d). The different processes taking place at interface for each electrode can be modeled by the construction of an electrical equivalent circuit (EEC). The EEC used to obtain electrochemical parameters is presented as inset in Figure SI 1d. This circuit includes the resistance of the electrolyte Rs, the charge transfer resistance Rct, the double layer capacity Cdl, and the Warburg impedance Zw (parameters related to the diffusion of electroactive species at the interface). The good correlation between fit data and experimental curves was checked with *χ*^2^ coefficient which is around 10^−3^ and has validated the EEC used. The highest Rct (53217.09 Ω.cm^2^) is obtained with CPE/NiAl, thus confirming a poor conductivity of LDH. The Rct value obtained with CPE/NiAl-DSS (Rct = 10133.17 Ω.cm^2^) is found to be 1.26 times smaller than that of the bare CPE (Rct = 12777.20 Ω.cm^2^). This result confirms that the presence of modifier (DSS) on CPE facilitates the electron exchange.

### 3.3. Application for the Electrochemical Determination of Fenuron

#### 3.3.1. Active Surface Area of the Working Electrode

The Randles–Sevcik equation was used to calculate the active surface area of electrodes [54, 55]. The area was measured using cyclic voltammetry at various sweep rates with 1 × 10^−4^ M K_3_[Fe(CN)_6_] solution in 0.1 M NaCl solution. The value of the area *A*_0_ was calculated using the slope of the plot of Ip versus *v*^1/2^ from (1) (Figure SI 2).

(1) $$\begin{array}{c} i_{p}=\left(2.69\times 10^{5}\right)n^{3/2}A_{0}D_{0}^{1/2}v^{1/2}C_{0}, \end{array}$$

where *C*_0_ is K_3_[Fe(CN)_6_] concentration 10^−4^ M, *n* is the number of electrons (*n* = 1) engaged in the process, *i*_*p*_ denoted anodic peak current, *D*_0_ is diffusion coefficient (7.610^−6^ cm^2^/s; [15]), *A*_0_ is the working electrode surface area, and *v* is the sweep rate. For bare CPE, CPE/NiAl-LDH, and CPE/NiAL-DSS, the effective surface areas were determined to be 0.048 cm^2^, 0.0529 cm^2^, and 0.0833 cm^2^, respectively.

#### 3.3.2. Fenuron Behaviour on Modified Electrodes

**Figure 3:**
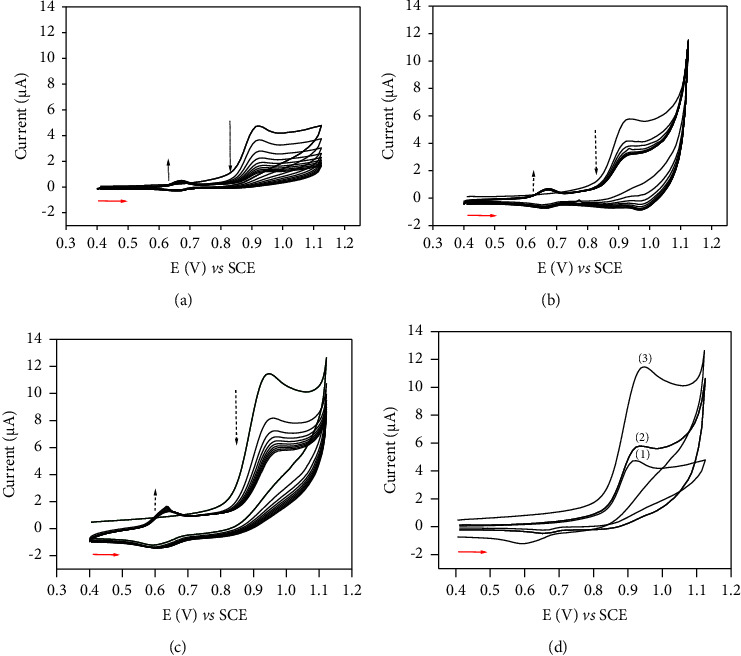
Multicyclic voltammograms of FEN 5 × 10^−5^ M recorded in acetate buffer solution 0.1 M (pH 4.7) at 50 mv/s on (a) CPE, (b) CPE/NiAl, and (c) CPE/NiAl-DSS. (d) Superimposition of the first scans recorded on CPE (1), CPE/NiAl (2), and CPE/NiAl-DSS (3).

Multicyclic voltammetry was conducted in 0.1 M acetate buffer to investigate the electrochemical behaviour of FEN on the CPE modified with the organo-LDH. The voltammograms of 5 × 10^−5^ M FEN on bare CPE or CPE modified with either LDH or its organically modified form is shown in Figure 2. The electrochemical response on bare CPE (Figure 3(a)) is composed of two redox systems: an irreversible anodic peak (*E*_pa_ = 0.92 V) and a pair of reversible peaks (*E*_pa_ = 0.68 V and *E*_pc_ = 0.67 V). As seen in the first scan, one anodic peak appears at 0.92 V, while when the scan direction is reversed, only one cathodic peak appears at 0.67 V. New anodic peak appears at 0.68 V on the anodic sweep in each successive cycle, forming a reversible system. It should be noted that the intensity of the peak current, which appears around 0.92 V after the first scan, decreases relatively slowly during the multiscan, indicating a low passivation of the electrode at this concentration [56]. The presence of the reversible system which is centered at 0.675 V depends on the oxidation product of FEN, which appears at 0.92 V. In fact, the reversible redox system did not appear when the potentials were scanned from 0.4 V to 0.7 V (Figure SI 3). This reversible system could be due to the presence of quinonic function generated on the aromatic ring after the first oxidation of FEN [56, 57]. Similar, multicyclic voltammetry was employed to compare the electrochemical behaviour of FEN on the CPE/NiAl and CPE/NiAl-DSS. The electrochemical behaviour of FEN on the CPE/NiAl and CPE/NiAl-DSS was similar to that seen on the bare CPE, as illustrated in Figure 3(b) and 3(c). However, there are some considerable changes. Firstly, the voltammogram obtained on CPE/NiAl (Figure 3(b)) showed from the second scan, a reduced shoulder peak which appears at 0.98 V, whose intensity increases with the number of scans. This shoulder could be attributed to the reduction of the degradation product after oxidation of FEN [56]. Secondly, on the voltammogram obtained on CPE/NiAl-DSS (Figure 3(c)), the system which appears around 0.68 V on CPE/NiAl shifts towards the cathodic potential for about 60 mV. Compared to CPE/NiAl-DSS (Figure 3(d)), the signals recorded during the first scan on bare CPE and CPE/NiAl are less intense. In fact, the current associated to the oxidation of FEN on CPE/NiAl-DSS was 2-fold and 2.7-fold more intense than that recorded on CPE/NiAl, and CPE, respectively. This advantageous effect is explained by the presence of the anionic surfactant (DSS). In fact, surfactants are known to exhibit a great adsorption capacity, and their intercalation in layers of LDH can make their surface hydrophobic. Such organo-LDHs are likely to sorb neutral hydrophobic molecules [32]. Because FEN is a highly organophilic compound, there will be a strong attraction (through van der Waals interactions) between FEN and the CPE/NiAl-DSS, which significantly increases the electrode surface's affinity for the FEN in the analytical solution.

#### 3.3.3. Optimization of Experimental Parameters for FEN Detection

**Figure 4:**
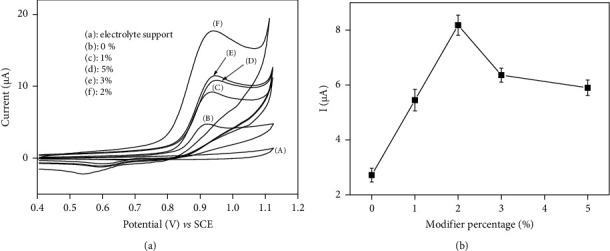
(a) Cyclic voltammograms of (A) 0.1 M acetate buffer solution (pH 4.7) on CPE/NiAl- DSS (2%); cyclic voltammograms of FEN 5 × 10^−5^ M recorded in acetate buffer solution 0.1 M (pH 4.7) at 50 mv/s on (B) CPE, (C) CPE/NiAl-DSS (1%), (D) CPE/NiAl- DSS (5%), (E) CPE/NiAl-DSS (3%), and (F) CPE/NiAl-DSS (2%); (b) variation of the current as a function of the percentage of NiAl-DSS in CPE.

*(1) The Effect of NiAl-DSS Amount on the Paste Composition*. It was anticipated that the electrode response would vary with the fraction of NiAl-DSS in the CPE. Before it, experimental parameters were carried out to ascertain the best condition (Table SI 5).  Figure 4(a) displays the cyclic voltammogram of FEN 5 × 10^−5^ M in 0.1 M acetate buffer on CPE/NiAl-DSS prepared with different quantities of NiAl-DSS in the paste. Up to a maximum of 2% (Figure 4(b)), the peak current intensity increases with increasing NiAl-DSS incorporation in the paste; however, as NiAl-DSS incorporation increases further, the peak current intensity decreases. The initial increase in the peak current was due to an increase in the number of FEN adsorption sites within the paste, while the subsequent loss of the peak current observed at NiAl-DSS proportions greater than 2% could be attributed to the fact that the proportion of NiAl-DSS in the composition of CPE is increasing, which contributed to reduce the electrode's conductivity [58]. The optimal percentage of NiAl-DSS incorporated into the CPE was determined to be 2%, representing the best compromise between the number of active sites and the paste's conductivity.

**Figure 5:**
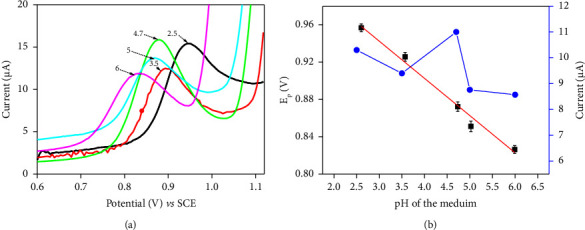
(a) Influence of the pH medium on the DPV response of FEN 5 × 10^−5^ M. (b) (blue line) effect of pH of medium on the anodic peak current of FEN on a CPE/NiAl-DSS and (red line) variation of the peak potential versus pH of medium.

*(2) Effect of pH on the FEN Signal*. The acidity of the medium is an important parameter that can influence mass transport on the electrode surface, particularly when the redox process involves proton transfer, as in this case. The DPV curves of FEN 5 × 10^−5^ M in acetate buffer 0.1 M on CPE/NiAl-DSS when the pH was varied from 2.5 to 6 are shown in Figure 5(a). The peak current and potential were found to vary with the pH of the electrolytic solution. The results in Figure 4(b) showed that the electrode's sensitivity increased in the pH range 3.5 < pH < 4.7 and then decreased with increasing solution pH and reaching optimal performance at pH 4.7. The low sensitivity obtained in the pH range 2.5 < pH < 3.5 can be attributed to metal ion hydrolysis and LDH instability [59]. The observed loss of sensitivity for pH values up to 4.7 may be explained by the fact that FEN was partially degraded in the pH range 5 to 6 [27]. As shown in Figure 5(a), the peak potential (*E*_*p*_) gradually shifted to cathodic values as the pH varied from 2.5 to 6. Peak potential as a function of electrolytic solution pH is plotted as a straight line (Figure 5(b)). The equation is *E*_*p*_ = −0.0311 pH + 1.0241 (*R*^2^ = 0.9869), with a slope of −0.0311 V/pH. According to the Nernst equation, the value of the slope (*p*) is given by equation (2). Here, *m* and *n* are, respectively, the number of protons and electrons exchanged per mole of substance. The slope found in this instance (31 mV per pH) shows that the *m*/*n* ratio is close to 1/2.

(2) $$\begin{array}{c} p=\frac{m}{n}\times 0.059. \end{array}$$

**Figure 6:**
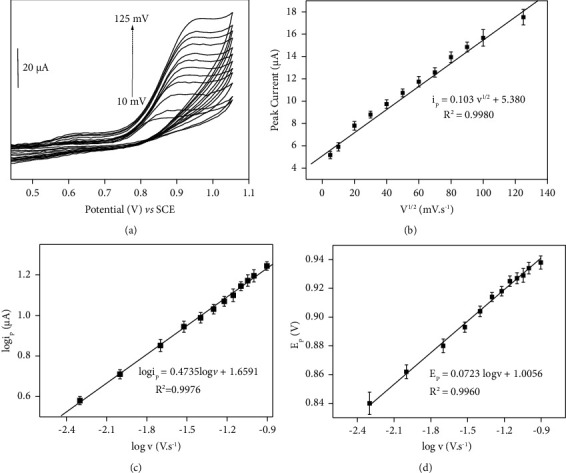
(a) Influence of the scan rate (*v*) on the peak current of 5 × 10^−5^ M FEN on CPE/NiAl-DSS (*v* = 25–125 mv.s^−1^). (b) Plot of the anodic peak current vs. *v*^1/2^. (c) Log (Ipa) vs. log (*v*). (d) Plot of variation of the peak potential with logarithm of the scan rate for the oxidation peak of FEN.

*(3) Electrochemical Oxidation Mechanism for FEN*. The impact of the scan rate on the FEN signal using cyclic voltammetry was investigated to provide further insight into the electrochemical behaviour of FEN at CPE/NiAl-DSS, as described in Figure 6(a). From this graph, we can see that the oxidation peak current increases with the scan rate. The linear relationship between the anodic peak current and the square root of the scan rate (Figure 6(b)) indicates that the electrochemical reaction at the clay-modified electrode is diffusion-controlled [60]. Additionally, the plot of logi_p_ against log *v* for the oxidation peak of FEN is linear, further supporting these findings (Figure 6(c)). The obtained slope value of 0.47, which is close to the theoretical value of 0.5, indicates that the oxidation of FEN occurring on the CPE/NiAl-DSS is primarily a diffusion-controlled process [60]. The data such as slope, intercept, and standard error of intercept of Figures 6(b), 6(c), and 6(d) are summarized in Tables SI 2, 3, and 4.

Furthermore, as depicted in Figure 5(a), there is a shift of peak potential (*E*_*p*_) of the oxidation peak towards more anodic values with the increase of the scan rate. Similarly, the plot of *E*_*p*_ versus log *v* is also linear (Figure 6(d)), which can be described as Laviron's equation (3) [61].

(3) $$\begin{array}{c} E_{p}=E^{0}+\left[\frac{2.303RT}{\alpha nF}\right]\log\left[\frac{{RTK}_{s}}{\alpha nF}\right]+\left[\frac{2.303RT}{\alpha nF}\right]logv, \end{array}$$

where *α* is the electron transfer coefficient, *n* is the electron-transfer number, *F* is the Faraday constant (96,480 C.mol^−1^), *E*_*p*_ is the oxidation peak potential (V), *E*^0^ is the formal potential, *R* is the universal gas constant (8.314 J mol^−1^.K), *T* is the temperature (298 K), and *v* is the scan rate (mV.s^−1^). Due to FEN's irreversible electrochemical behaviour, the value of *α* was considered to be 0.5 [54, 62]. As a result, the number of electrons calculated was 1.64 electrons, which is close to 2. We can therefore deduce that the electrochemical reaction's electron number *n* and proton number *m* are 2 and 1, respectively, based on the results of the pH effect. These results are consistent with the earlier study and shows that one proton and two electrons are required for the oxidation of FEN, with the nitrogen atom in the amide group most likely to be oxidized [3, 63]. The following equation can be used to describe the oxidation pathway of FEN.(4)
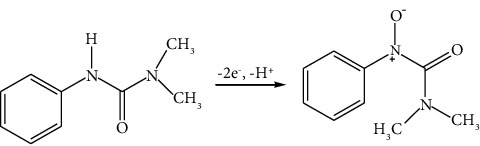


*(4) Repeatability and Stability of FEN on CPE/NiAl-DSS*. The objective of this study is to develop a sensitive carbon paste electrode for the electrochemical detection of FEN. To assess the sensor's performance, the repeatability and stability were evaluated. In this regard, seven consecutives measurements were conducted using the same electrode for the detection of 5 × 10^−5^ M of FEN solution (Figure SI 4). The seven parallel responses show almost the same peak current value (RSD = 3%). This result indicates that the signal of FEN is stable on CPE/NiAl-DSS. Further, stability of CPE/NiAl-DSS was investigated by measuring the response of 5 × 10^−5^ M of FEN every day during five days. After each recording and desorption, the sensor was kept in a refrigerator in a 0.1 M acetate buffer. After 5 days, the current response decreased by only 5% from its initial value, indicating good stability over time.

**Figure 7:**
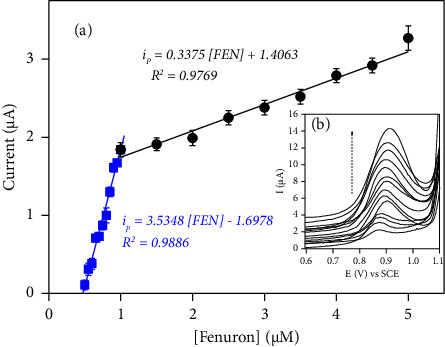
(a) Calibration plots obtained for FEN sensing at carbon paste modifier with NiAl-DSS (prepared with 2% of NiAl-DSS in the composition of the paste) in the concentration range 0.5 × 10^−6^ to 1 × 10^−6^ mol/L and 1 × 10^−6^ to 5 × 10^−6^ mol/L in 0.1 M acetate buffer solution (pH 4.7). (b) Inset DPV curves recorded.

*(5) The Effect of FEN Concentration*. The optimum settings outlined above were used to study the impact of analyte concentration.  Figure 7(b) shows a typical DPV peak for a pesticide solution produced at concentration ranges of 0.5 × 10^−6^ to 1 × 10^−6^ mol/L and 1 × 10^−6^ to 5 × 10^−6^ mol/L in 0.1 M acetate buffer solution (pH 4.7). The current vs. FEN concentration curve (Figure 7(a)) exhibited wide linearity. The detection limit (3 S/n) and quantification limit (10 S/n) were found to be 1.8 × 10^−8^ mol.L^−1^ and 6 × 10^−8^ mol.L^−1^, respectively, with respect to the calibration graph's standard deviation (SD) of intercept and slope value of calibration curve (in a low concentration range). The observed sensitivity at low concentration was 3.53 *μ*A/*μ*M, which is 10 times higher than the value attained at high concentration. As a result of the optimization process, the most sensitive peak recorded and instrumental parameters for FEN determination are summarized in Tables SI 5 and SI 6, respectively.  Table 2 compares the performance of CPE/NiAl-DSS with that reported in the literature, including the limit of detection and the linear range, and it shows that the proposed sensor had detection limits that were lower than those reported by some authors for trace analyses of some phenylurea pesticides [3, 29, 30, 64, 66, 67].

*(6) Interferences and Analysis of Real Samples*. The selectivity of this method for the determination of FEN was evaluated by introducing other ions likely to be found in a natural water sample. To this end, known amounts of these ions were added to a solution containing 5 × 10^−5^ M of FEN. The resulting solutions were then analyzed, and the results obtained are summarized in Table 3. It can be seen on this table that no significant effect on the FEN response was observed even in the presence of Na^+^, K^+^, Ca^2+^, Cl^−^, SO_4_^2−^, and Mg^2+^ excesses up to 1000-fold. However, Pb^2+^ and Zn^2+^ at concentrations 500 times greater than FEN are significantly interfered by reducing the intensity of FEN. Additionally, methyl parathion and glyphosate strongly interfered with the determination of FEN, which could compromise the method's selectivity. Therefore, we recommend the removal of these interfering species (Pb^2+^, Zn^2+^, glyphosate, and methyl parathion) from matrices prior to FEN quantification. Actually, it has been documented that an inorganic ion like Pb^2+^ can be eliminated from a medium by applying a common chelating agent like ethylene diamine tetraacetic acid (EDTA) [72–74]. Smectite that has been treated by organosilane can be used to remove glyphosate [75] and methyl parathion [76].

The sensor developed under ideal circumstances was utilized to identify FEN in different samples of water collected in Yaoundé town. In practice, a sufficient volume of this sample was used to prepare the electrolytic solution (acetate buffer), and following analyses under the optimum circumstances for FEN detection, no signal was recorded utilizing the DPV technique. The amounts recovered if we spiked this sample water with a typical FEN solution are listed in Table 4. This finding suggests that the sensor elaborated can be effectively used for the detection of FEN in a practical setting.

## 4. Conclusion

In this study, it was demonstrated that a NiAl-LDH intercalated by an anionic surfactant served as a valuable material for the development of an amperometric sensor for FEN. Before the use of this organo-LDH for sensing of FEN, infrared spectroscopy, X-ray diffraction, and thermal analysis results confirmed the intercalation of the surfactant. This organo-LDH was added to the carbon paste electrodes, which increased the peak current and enhanced electrode sensitivity. When compared to the response of the same electrode changed by pristine LDH, the oxidation peak of FEN was enhanced (2-fold). A detection limit of 1.8 × 10^−8^ mol L^−1^ was reached after optimization. The proposed method was used to calculate the amount of FEN in river water, showing that it has potential for monitoring FEN in diverse polluted media.

## Figures and Tables

**Table 1:** EDS analysis of the samples.

Samples	Element % by mass^a^						
	C	N	O	Na	S	Ni	Al
LDH	4.49	15.34	34.73	37.46	—	5.97	2.01
LDH-DSS	8.12	—	40.23	0.25	4.8	35.1	11.5

^a^Each value is an average taken over 3 different spots on the samples.

**Table 2:** Comparison of the proposed method with literature methods for the determination of phenyl urea herbicides.

Electrode configuration	Linearity range (M)	Detection limit (M)	Analyte	Reference
GO–MWCNTs/ ^*∗*^GCE	0.30 × 10^−6^–15 × 10^−6^	0.10 × 10^−6^	Isoproturon	[3]
GO–MWCNTs/GCE	1 × 10^−6^–47 × 10^−6^	1.5 × 10^−6^	Fenuron	[29]
GO–MWCNTs/ ^*∗*^GCE	1 × 10^−6^–47 × 10^−3^	0.35 × 10^−6^	Fenuron	[30]
Polypyrrole/ ^*∗*^GCE	0.001 × 10^−6^–110 × 10^−6^	0.005 × 10^−6^	Fenuron	[31]
PtNPs/CS/ ^*∗*^GCE	0.195 × 10^−6^ –4.847 × 10^−6^	0.034 × 10^−6^	Isoproturon	[63]
FeTAPc-SWCNT/ ^*∗*^GCE	5 × 10^−5^–1 × 10^−4^	2.7 × 10^−7^	Diuron	[64]
Ph-CN-SWCNT/ ^*∗*^GCE	1 × 10^−5^−2 × 10^−4^	0.18 × 10^−6^	Diuron	[65]
NiTAPc/ ^*∗*^GCE	3×10^−5^–3.5 × 10^−4^	3.3 × 10^−7^	Diuron	[66]
^*∗∗*^CPE	0.12 × 10^−4^−2.5 × 10^−4^	2 × 10^−6^	Diuron	[67]
GO–MWCNTs/ ^*∗*^GCE	0.215 × 10^−6^−2.15 × 10^−6^	6.88 × 10^−8^	Diuron	[68]
Organo-Montmorillonite/ ^*∗*^GCE	4.84 × 10^−9^–1.45 × 10^−6^	4.84 × 10^−9^	Isoproturon	[69]
PANI/MWCNTs/ ^*∗*^GCE	4.84 × 10^−8^–4.84 × 10^−4^	4.84 × 10^−10^	Isoproturon	[70]
Graphene modified/ ^*∗*^GCE	9.69 × 10^−8^–4.84 × 10^−5^	9.69 × 10^−8^	Isoproturon	[71]
NiAl-DSS/^*∗∗*^CPE	0.5 × 10^−6^–1 × 10^−6^	1.8 × 10^−8^	Fenuron	This study

^*∗*^GCE: glassy carbon electrode, ^*∗∗*^CPE: carbon paste electrode GO-MWCNT: graphene-multiwalled carbon nanotubes. PANI/MWCNTs: polyaniline multi-walled carbon nanotubes PtNPs/CS: platinum/chitosan nanoparticle. Ph-CN-SWCNT: phthalocyanine-single-walled carbon nanotube FeTAPc: iron (II) tetraaminophthalocyanine. NiTAPc: polymerized nickel tetraaminophthalocyanine.

**Table 3:** Effect of interfering analytes for detection of FEN 5 × 10^−5^ M on CPE/NiAl-DSS.

Interference ions	Added amount over (FEN)	Percentage signal variation
Cl^−^	1000	0
SO_4_^2−^	1000	0
Na^+^	1000	0
Mg^2+^	1000	0
Zn^2+^	500	−16.6
Pb^2+^	500	−20
Methyl parathion	10	−7
Glyphosate	10	−15

**Table 4:** Determination of FEN in water.

	FEN added (*µ*M)	FEN found (*µ*M)^a^	Recovery (%)
River water	0.85	(0.83 ± 0.01)	97.65
	1	(0.98 ± 0.03)	98

Tap water	1	(0.96 ± 0.02)	96

Ground water	1	(0.99 ± 0.05)	99

^a^Number of samples assayed = 5.

## Data Availability

The data used to support the fndings of this study are included within the article.

## Supplementary Materials

Supplementary Materials Figure SI 1: multicyclic voltammograms of 10−4 M [Fe(CN)6]3-recorded in 0.1 M NaCl on (a) bare CPE, (b) CPE/NiAl-LDH, and (c): CPE/NiAlLDH-DSS. (d) Superposition of experimental and fitted Nyquist data recorded on CPE, CPE/NiAl-LDH, and CPE/NiAlLDH-DSS in 0.1 M NaCl containing 10^−4^ M [Fe(CN)_6_]^3−/4−^. Inset figure is the equivalent circuit model used for fitting. Figure SI 2: linear relation of the anodic peak current against the square root of scan rate of 10−3 M of [Fe(CN)6]3-in NaCl 0.1 (M) (a): bare CPE, (b): CPE/NiAl-LDH, and (c): CPE/NiAlLDH-DSS. Inset the corresponding CVs (a): bare EPC, (b): CPE/NiAl-LDH, and (c): CPE/NiAlLDH-DSS in 10−4 M [Fe(CN)6]3-at different scan rates from 10 to 60 mV.s^−1^. Figure SI 3: (A): cyclic voltammograms of FEN 5×0–5 M recorded in acetate buffer solution 0.1 M (pH 4.7) at 50 mv/s on CPE/NiAl-DSS (a) from 0.4 V to 0.7 V and (b) from 0.4 V to 1.1 V; (B) cyclic voltammograms of FEN on CPE/NiAl-DSS from 0.65 V to 1.1 V in a same solution. Figure SI 4: typical successive peaks recorded using 5 × 0–5 M of FEN in 0.1 M acetate buffer (pH 4.7) on CPE/NiAl-DSS; inset DPV response recorded. Table SI 1: assignment of FT-IR band in LDH and organo-LDH. Table SI 2: linear data of figure 6B. Table SI 3: linear data of figure 6C. Table SI 4: linear data of figure 6D. Table SI 5: regression data of the calibration curve for the DPV voltammetric determination of FEN. Table SI 6: optimum instrumental parameters for the DPV voltammetric determination of FEN.
