# Supplementary material for: Dioctylsulfosuccinate Functionalized NiAl-Layered Double Hydroxide for Sensitive Fenuron Electroanalysis Using a Carbon Paste Electrode
Source: J Anal Methods Chem. 2024 Jul 19;2024:9237309. doi: 10.1155/2024/9237309 (PMC11458295; doi:10.1155/2024/9237309)
Supplement: Supplementary Materials — Figure SI 1: multicyclic voltammograms of 10−4 M [Fe(CN)6]3-recorded in 0.1 M NaCl on (a) bare CPE, (b) CPE/NiAl-LDH, and (c): CPE/NiAlLDH-DSS. (d) Superposition of experimental and fitted Nyquist data recorded on CPE, CPE/NiAl-LDH, and CPE/NiAlLDH-DSS in 0.1 M NaCl containing 10−4 M [Fe(CN)6]3−/4−. Inset figure is the equivalent circuit model used for fitting. Figure SI 2: linear relation of the anodic peak current against the square root of scan rate of 10−3 M of [Fe(CN)6]3-in NaCl 0.1 (M) (a): bare CPE, (b): CPE/NiAl-LDH, and (c): CPE/NiAlLDH-DSS. Inset the corresponding CVs (a): bare EPC, (b): CPE/NiAl-LDH, and (c): CPE/NiAlLDH-DSS in 10−4 M [Fe(CN)6]3-at different scan rates from 10 to 60 mV.s−1. Figure SI 3: (A): cyclic voltammograms of FEN 5×0–5 M recorded in acetate buffer solution 0.1 M (pH 4.7) at 50 mv/s on CPE/NiAl-DSS (a) from 0.4 V to 0.7 V and (b) from 0.4 V to 1.1 V; (B) cyclic voltammograms of FEN on CPE/NiAl-DSS from 0.65 V to 1.1 V in a same solution. Figure SI 4: typical successive peaks recorded using 5 × 0–5 M of FEN in 0.1 M acetate buffer (pH 4.7) on CPE/NiAl-DSS; inset DPV response recorded. Table SI 1: assignment of FT-IR band in LDH and organo-LDH. Table SI 2: linear data of figure 6B. Table SI 3: linear data of figure 6C. Table SI 4: linear data of figure 6D. Table SI 5: regression data of the calibration curve for the DPV voltammetric determination of FEN. Table SI 6: optimum instrumental parameters for the DPV voltammetric determination of FEN. [file 9237309.f1.zip › Table SI 5.docx.pdf]

Table SI 5: Regression data of the calibration curve for the DPV voltammetric determination of FEN

Parameter (Unit)	Values
Linearity range (mol/L)	0.5×10^{-6}-1×10^{-6}
Slope (μA L/mol)	3.53 ± 0.13
Intercepts (μA)	1.69 ± 0.1073
Correlation coefficient	0.9886
Limit of determination (LOD) (mol/L)	1.8×10^{-8}
Limit of quantification (LOQ) (mol/L)	6×10^{-8}
